# Efficacy and Safety of Raltegravir-Based Dual Therapy in AIDS Patients: A Meta-Analysis of Randomized Controlled Trials

**DOI:** 10.3389/fphar.2019.01225

**Published:** 2019-10-17

**Authors:** Yinqiu Huang, Xiaojie Huang, Hui Chen, Hao Wu, Yaokai Chen

**Affiliations:** ^1^National Key Laboratory for Infectious Diseases Prevention and Treatment with Traditional Chinese Medicine, Chongqing Public Health Medical Center, Chongqing, China; ^2^Center for Infectious Diseases, Beijing You’an Hospital, Capital Medical University, Beijing, China; ^3^School of Biomedical Engineering, Capital Medical University, Beijing, China; ^4^Department of Infectious Diseases, Chongqing Public Health Medical Center, Chongqing, China

**Keywords:** HIV, raltegravir, protease inhibitor, efficacy and safety, simplified regimen, meta-analysis

## Abstract

**Background:** The life expectancy for HIV-infected individuals has improved dramatically because of improvements in antiretroviral therapy (ART). Today, a simplified two-drug regimen enhances adherence and treatment satisfaction by reducing adverse effects. Therefore, we need more evidence to show the benefits and risks of simplified ART regimens from randomized controlled trials (RCTs). We compared the efficacy and safety of raltegravir-based simplified dual therapy (DT) and of traditional triple therapy (TT) for people living with HIV/AIDS (PLWHA).

**Methods:** We carried out a systematic review of RCTs. After using a combination of the key words “HIV,” “raltegravir,” and “protease inhibitor” to search the English-language electronic databases from January 1, 2004, to September 11, 2019, we pooled data across eligible studies and estimated the summary effect sizes with Review Manager (version 5.3).

**Results:** We included eight RCTs involving 4420 PLWHA: 2187 (49.5%) received raltegravir-based simplified DT, and 2144 (48.5%) received traditional TT. The proportion of viral suppression was 79% at 48 weeks and 74% at 96 weeks in the simplified regimen, and the proportion of viral suppression was 78% at 48 weeks and 71% at 96 weeks in the traditional TT group. Furthermore, the proportion of viral suppression in the simplified DT group was greater than that in the TT group at 24 weeks (risk ratio 1.11, 95% confidence interval 1.02-1.21; p = 0.01). The CD4 cell counts in the simplified DT group were significantly higher at 48 weeks and 96 weeks than those in the group that received the traditional TT. Regarding adverse events and mortality rates, the DT and TT groups were similar. However, there was better adherence in the DT group than in the TT group.

**Conclusion:** We found that the simplified regimen was noninferior to TT regimen in regard to viral suppression. Furthermore, the simplified DT regimen had a better CD4 cell count and lower adverse events than the TT regimen.

## Introduction

The expansion of access to antiretroviral therapy (ART) has averted millions of deaths for those who are infected with HIV (Gilks et al., 2006; WHO, 2013b). In the current World Health Organization (WHO) guidelines, the recommended initial therapy for patients infected with HIV-1 is a combination ART that includes two nucleoside reverse-transcriptase inhibitors (NRTIs) and nonnucleoside reverse-transcriptase inhibitors (NNRTIs) or a ritonavir-boosted PI (PI/r) (WHO, 2016). When NRTIs are used in first-line or second-line therapy regimens, the tolerability, safety, and toxicity profiles are limiting. For example, the combination of tenofovir (TDF) and emtricitabine (FTC) could cause renal and bone complications (McComsey et al., 2010; Hall et al., 2011; Brown, 2013). These shortcomings have led to research on alternative combinations without NRTIs to expand treatment options. The WHO’s proposed public health approach provides treatment for millions of people in low-income and middle-income countries with weak health systems (WHO, 2010 revision. http://whqlibdoc.who.int/publications/2010/9789241599764_eng.pdf (accessed April 1, 2013)]. This approach emphasizes the importance of simple, effective, safe, and tolerable treatment that can be performed by trained, non-health-care workers in accordance with simple procedures. In various formulations, a simplified approach can also reduce the need for multiple drug stocks (Group et al., 2013). Some trials have found the efficacy and safety of ritonavir-boosted lopinavir (LPV/r) plus raltegravir (RAL) to be largely similar to that of LPV/r plus NRTIs (Gallien et al., 2011; Nguyen et al., 2011a; Reynes et al., 2013). RAL has been increasingly used clinically in first-line and second-line ART regimens because it is more efficacious and better tolerated than NRTIs.

RAL was launched in 2007 as a first integrase inhibitor (Croxtall and Scott, 2010; Nguyen et al., 2011b) it was efficacious and generally well tolerated in patients infected with HIV-1 (Markowitz et al., 2007; Cooper et al., 2008; Lennox et al., 2010) and could serve as the third drug in a combination ART regimen (Church, 2009; Lennox et al., 2009). HIV infection is a chronic disease, and affected patients will receive life-long therapy. In treatment-experienced patients with multidrug-resistant HIV compared with patients who receive placebo plus optimized background therapy (OBT), RAL with OBT has been shown to be well tolerated, safe, and effective in producing viral suppression in treatment-experienced patients at 16 and 24 weeks (Grinsztejn et al., 2007). PI/r plus RAL was included as an alternative regimen in the 2016 WHO treatment guidelines (WHO, 2016). The WHO-preferred PI/r drugs are atazanavir, lopinavir, and darunavir on the basis of efficacy and tolerability (WHO, 2013a). Although increasing clinical evidence suggests that RAL plus PIs/r is an effective regimen (Raffi et al., 2013) to our knowledge, no meta-analysis has quantified the efficacy and safety of RAL plus PI/r compared with the efficacy and safety of two NRTIs plus PI/r.

This study aims to systematically evaluate the efficacy and safety of RAL-based simplified dual therapy (DT) in ART-naive and ART-experienced patients. We reviewed the literature to estimate differences in viral suppression, CD4 counts, adverse events, mortality, and adherence. These results will provide evidence-based recommendations for AIDS therapy.

## Methods

This review was reported according to the Preferred Reporting Items for Systematic Reviews and Meta-Analyses (PRISMA) statement (Moher et al., 2010), registration number CRD42017082468 (https://www.crd.york.ac.uk/prospero/display_record.php?ID=CRD42017082468).

### Data Sources

Systematic searches included all of the literature regarding RAL published in PubMed, MEDLINE, Web of Science, and Embase from January 1, 2004, to September 11, 2019. We searched for clinical trials using the following terms: “HIV,” “integrase inhibitors,” and “protease inhibitor”; only studies published in English were considered.

### Literature Inclusion and Exclusion Criteria

Only randomized controlled trials (RCTs) (n > 10) were included in the meta-analysis, and all patients had been treated with PIs/r with RAL or PIs/r with two NRTIs. The included studies incorporated treatment-experienced adults and adolescents with HIV who had failed a WHO-recommended first-line NRTI-based regimen and switched to an RAL-based simplified regimen. We excluded the following: (1) reviews, letters, case observations studies, and retrospective studies; (2) animal and *in vitro* experiments; (3) HIV-1 patients who were younger than 12 years old or pregnant; and (4) studies not including baseline CD4 cell counts or viral load monitoring.

### Study Selection and Exclusion Processes

Two investigators (YH and XH), working independently, scanned all titles and abstracts and excluded irrelevant articles. When divergence between the two investigators occurred, YC or HW arbitrated the dispute. Two investigators assessed the eligibility of full-text papers according to the inclusion and exclusion criteria. Then, data on the article’s characteristics, interventions at baseline, HIV RNA loads, CD4 cell counts, grade 3 or 4 adverse events, adherence, mortality, and drug resistance were independently extracted from the final list of selected eligible studies. The outcomes were chosen according to the WHO guidelines (WHO, 2016) and included viral suppression, the mean change in CD4 cell counts, grade 3 or 4 adverse events, drug resistance, mortality, and adherence. Any discrepancies between the investigators were resolved through discussion, and Dr. Chen arbitrated the dispute until a consensus was reached.

### Study Quality Assessment

The methodological quality of the RCTs was assessed by the Cochrane risk of bias tool; there were seven domains (Higgins et al., 2011). Study quality was recorded as “high risk,” “unclear risk,” or “low risk.” Studies meeting all criteria were considered to have a low risk of bias, whereas those meeting none of the criteria were considered to have a high risk of bias. Otherwise, studies were considered to have an unclear risk of bias.

### Statistical Analysis

Statistical analyses were performed with RevMan 5.3 software (The Nordic Cochrane Centre, The Cochrane Collaboration, Copenhagen, 2014). Dichotomous and continuous data are expressed as risk ratios (RRs) and mean differences (MDs), respectively, with 95% confidence intervals (95% CIs). Statistical heterogeneity was assessed by Cochrane’s Q test. If the heterogeneity test result was P ≥ 0.10 and I^2^ ≤ 50%, the studies were considered homogenous, and the fixed effect model was selected. In contrast, studies that were not homogeneous were assessed using a random-effects model.

## Results

### Characteristics of the Included Studies

**Figure 1:**
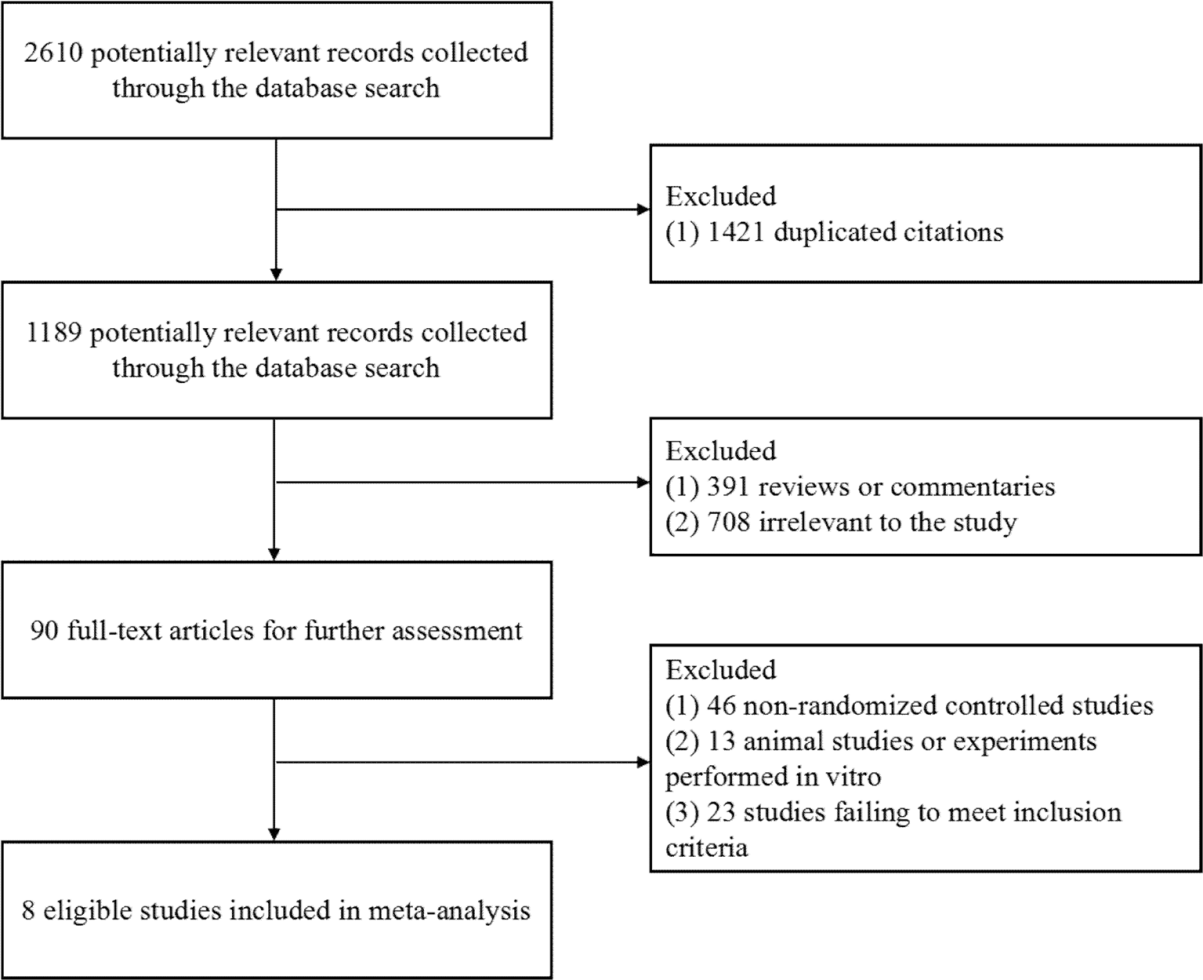
Flow diagram of the study selection. As shown, our initial searches yielded 2,610 records. The full texts of 1,189 articles were retrieved for detailed assessment after exclusion. Of these, 1,181 studies were subsequently excluded because they failed to meet the inclusion criteria; eight eligible studies were identified.

A total of 2,610 publications from four databases were identified by the initial screening; of these, Eight eligible articles were included in this meta-analysis (Reynes et al., 2011; Kozal et al., 2012; Group et al., 2013; Paton et al., 2014; Raffi et al., 2014; Amin et al., 2015; La Rosa et al., 2016; Hakim et al., 2018) (**Figure 1**). These trials were published from 2011 to 2018. Of these eight articles, six examined treatment with a combination of RAL and LPV/r, One examined treatment with RAL in combination with atazanavir/ritonavir (ATV/r), and one examined treated with a combination of RAL and darunavir/ritonavir (DRV/r) (**Table 1**). In this analysis, we included ART-naive patients and ART-experienced patients.

**Table 1:** Characteristics of the included studies.

Reference	Patient age	Cases	Interventions						Treatment (weeks)	Patients
			Treatment group			Control group				
			Regimes	Viral suppression rate	ΔCD4 from baseline (mean, SD/SE, range)	Regimens	Viral suppression rate	ΔCD4 from baseline (mean, SD/SE, range)		
Kozal et al. (2012)	≥18	94	RAL+ATV/r	74.6%	166 ± 133.9	ATV/r+RTV+TDF/FTC	63.3%	127 ± 95.9	24	ART-naive patients
Reynes et al. (2011)	≥18	206	RAL +LPV/r	83.2%	214.9	LPV/r+TDF/FTC	84.8%	245	4	ART-naive patients
Paton et al. (2014)	> 12	859	RAL +LPV/r	66.1%	260.0 ± 185.5	LPV/r+NRTIs	65.0%	234.0 ± 208.1	96	ART-experienced patients
Amin et al. (2015)	≥16	541	RAL +LPV/r	70.0%	228.7 ± 184.6	LPV/r+NRTIs	67.5%	201.7 ± 193.7	96	ART-experienced patients
Group et al. (2013)	≥16	541	RAL +LPV/r	71.1%	167.4 ± 142.1	LPV/r+NRTIs	70.5%	132.5 ± 146.0	48	ART-experienced patients
La Rosa et al. (2016)	≥18	515	RAL +LPV/r	82.3%	199.0 ± 131.2	LPV/r+NRTIs	80.4%	190.0 ± 133.6	48	ART-experienced patients
Raffi et al. (2014)	≥18	805	RAL+DRV/r	84.8%	266(250-283)	DRV/r +TDF–FTC	80.9%	268(250-284)	96	ART-naive patients
Hakim et al. (2018)	> 12	859	RAL +LPV/r	72.0%	296.0 ± 258.9	LPV/r+NRTIs	75.0%	290.0 ± 247.6	24	ART-experienced patients

RAL, raltegravir; LPV/r, ritonavir-booted lopinavir; NRTI, nucleoside reverse transcriptase inhibitor; DRV/r, ritonavir-booted darunavir; ATV/r, ritonavir-booted atazanavir.

### Methodological Quality of the Included Trials

**Figure 2:**
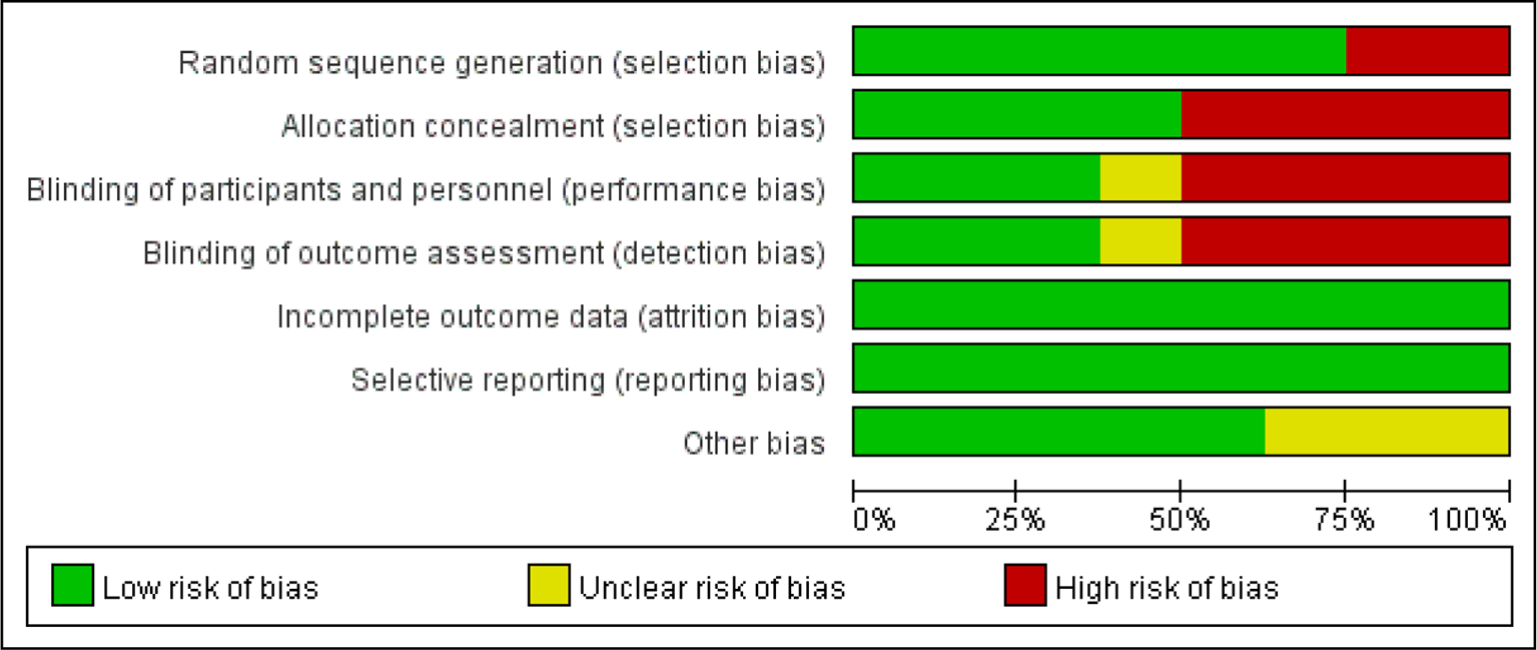
Quality assessment of the studies selected for systematic review. The risk of bias was used to assess the quality of the randomized controlled trials, and the majority of studies were found to be of low quality.

These articles are all based on randomized, open-label, noninferiority, and multicenter trials. According to the Cochrane risk of bias estimation, randomized participant allocation was mentioned in all trials; six trials (Reynes et al., 2011; Group et al., 2013; Paton et al., 2014; Raffi et al., 2014; Amin et al., 2015; Hakim et al., 2018) used a specific method, whereas two trials (Kozal et al., 2012; La Rosa et al., 2016) were defined as “high risk” for using an unclear randomization method. Three trials (Group et al., 2013; Raffi et al., 2014; Amin et al., 2015) blinded the participants and investigators to the treatment, four trials (Kozal et al., 2012; Paton et al., 2014; La Rosa et al., 2016; Hakim et al., 2018) only blinded the participants to the treatment, and one trial (Reynes et al., 2011) did not mention blinding. All trials reported complete outcome data, and there was no selective reporting. Five trials (Group et al., 2013; Paton et al., 2014; Raffi et al., 2014; La Rosa et al., 2016; Hakim et al., 2018) were low risk in terms of other biases (**Figure 2**).

### Outcome Measures

#### Plasma HIV-1 RNA Viral Load

**Figure 3:**
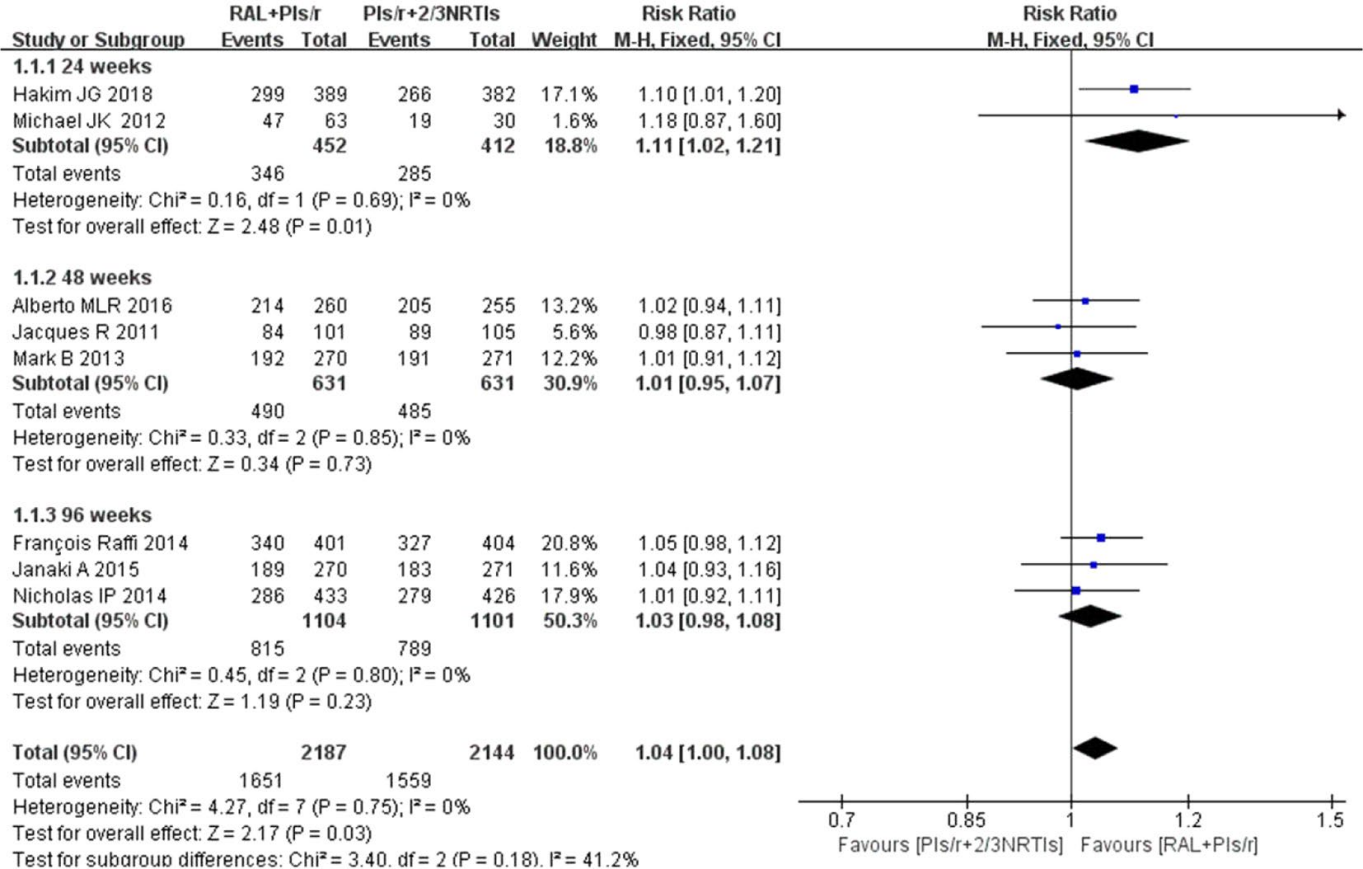
Forest plot comparing viral suppression with the two therapy regimens. (1) RAL = raltegravir. PIs/r = ritonavir-boosted protease inhibitor. NRTI = nucleoside or nucleotide reverse transcriptase inhibitor. (2) Study item displayed as the first author with the publication year. (3) I^2^ and P are the criteria of the heterogeneity test, with the —◆—pooled odds ratio, —¦— odds ratio and 95% confidence interval.

The results of the meta-analysis in terms of the viral suppression with RAL plus PIs/r (DT group) versus PIs/r plus two NRTIs (TT group) indicated comparable effects of the different regimens (viral suppression using 50 copies per ml). Two studies (Kozal et al., 2012; Hakim et al., 2018) that included 864 participants reported outcomes and indicated a significant difference between the two regimes at 24 weeks. The effect of DT was greater than that of TT (P = 0.69, I^2^ = 0%) [risk ratio 1.11, 95% CI [1.02, 1.21], P = 0.01] (**Figure 3**). The average viral suppression rate in the TT group was 69% (95% CI: 65%–74%), whereas it was 77% (95% CI: 73%–80%) in the DT group (**Table 1**). At 48 weeks and 96 weeks, the efficacy in viral suppression was not different between the TT and DT groups (risk ratio 1.01, 95% CI 0.95–1.07, P = 0.73; risk ratio 1.03, 95% CI 0.98–1.08, P = 0.23, respectively) (**Figure 3**). The viral suppression rate in the TT group was 78% at 48 weeks and 71% (95% CI: 61%–82%) at 96 weeks, whereas the viral suppression rate in the DT group was 79% (95% CI: 71%–87%) at 48 weeks and 74% (95% CI: 61%–86%) at 96 weeks (**Table 1**). A funnel plot was used to express the publication bias. The plots were symmetrical, suggesting that there was no obvious publication bias.

#### CD4 Cell Counts

**Figure 4:**
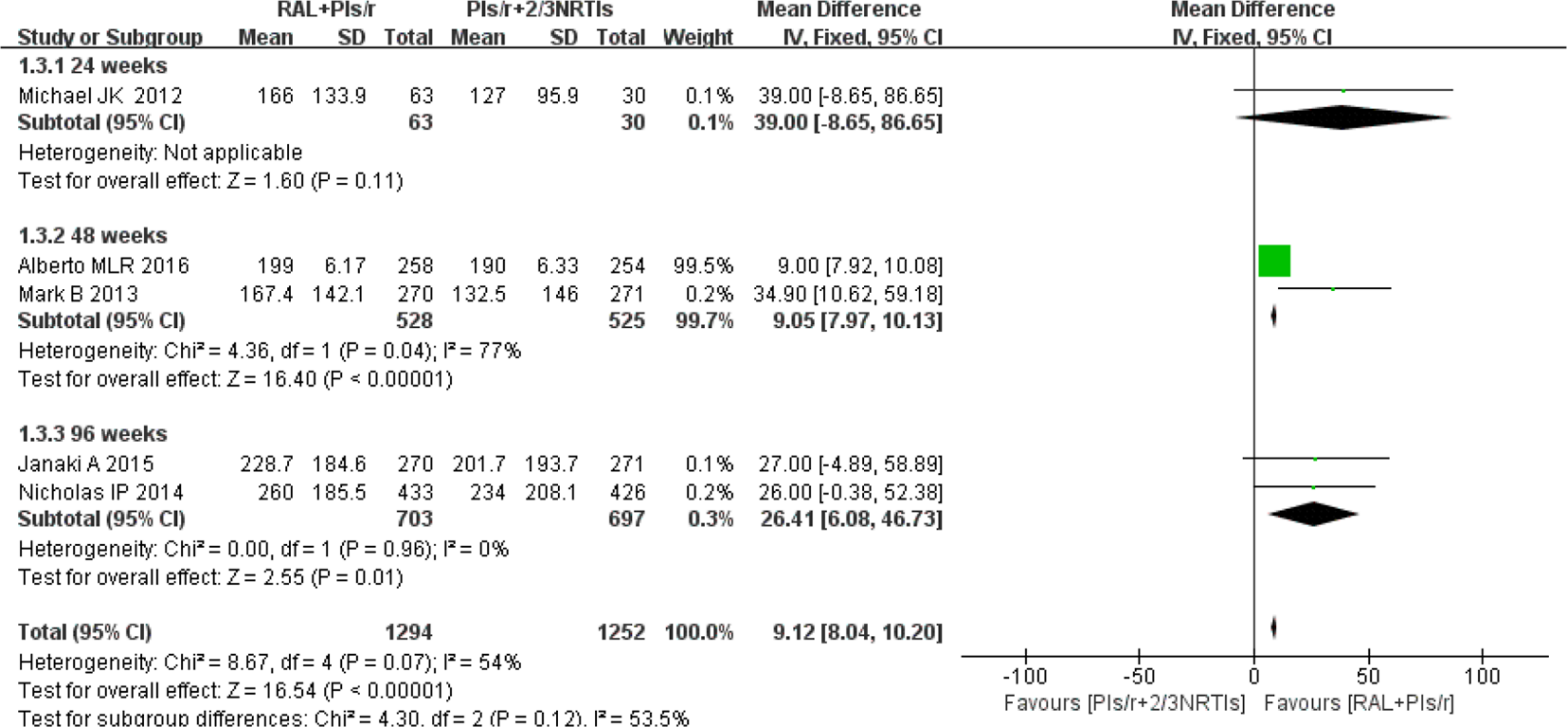
Forest plot comparing CD4 cell counts with simplified and traditional treatment therapy in HIV-1 patients. (1) RAL = raltegravir. PIs/r = ritonavir-boosted protease inhibitor. NRTI = nucleoside or nucleotide reverse transcriptase inhibitor. (2) Study item displayed as the first author with the publication year. (3) I^2^ and P are the criteria of the heterogeneity test, with the —◆—pooled odds ratio, —¦— odds ratio and 95% confidence interval.

Among the eight studies, five studies (Reynes et al., 2011; Group et al., 2013; Paton et al., 2014; Amin et al., 2015; La Rosa et al., 2016) contributed to the CD4 cell counts analysis. One (Reynes et al., 2011) of the five studies used the median to express the average CD4 cell counts. The findings showed that increases in mean CD4 counts were significantly higher in the DT group than in the TT group at 48 weeks and 96 weeks (mean difference 9.05, 95% CI 7.96–10.13, P < 0.01; mean difference 9.12, 95% CI 8.04–10.20, P = 0.01, respectively). This strategy showed no significant heterogeneity when the DT group was compared with the TT group at 96 weeks(P = 0.96, I^2 ^= 0%). However, at 48 weeks, there was significant heterogeneity between the two groups (P = 0.04, I^2 ^= 77%). In short, compared with the current standard of therapy regimen, simplified therapy showed a significant efficacy in terms of immune reconstruction (**Figure 4**).

#### Adverse Events

**Figure 5:**
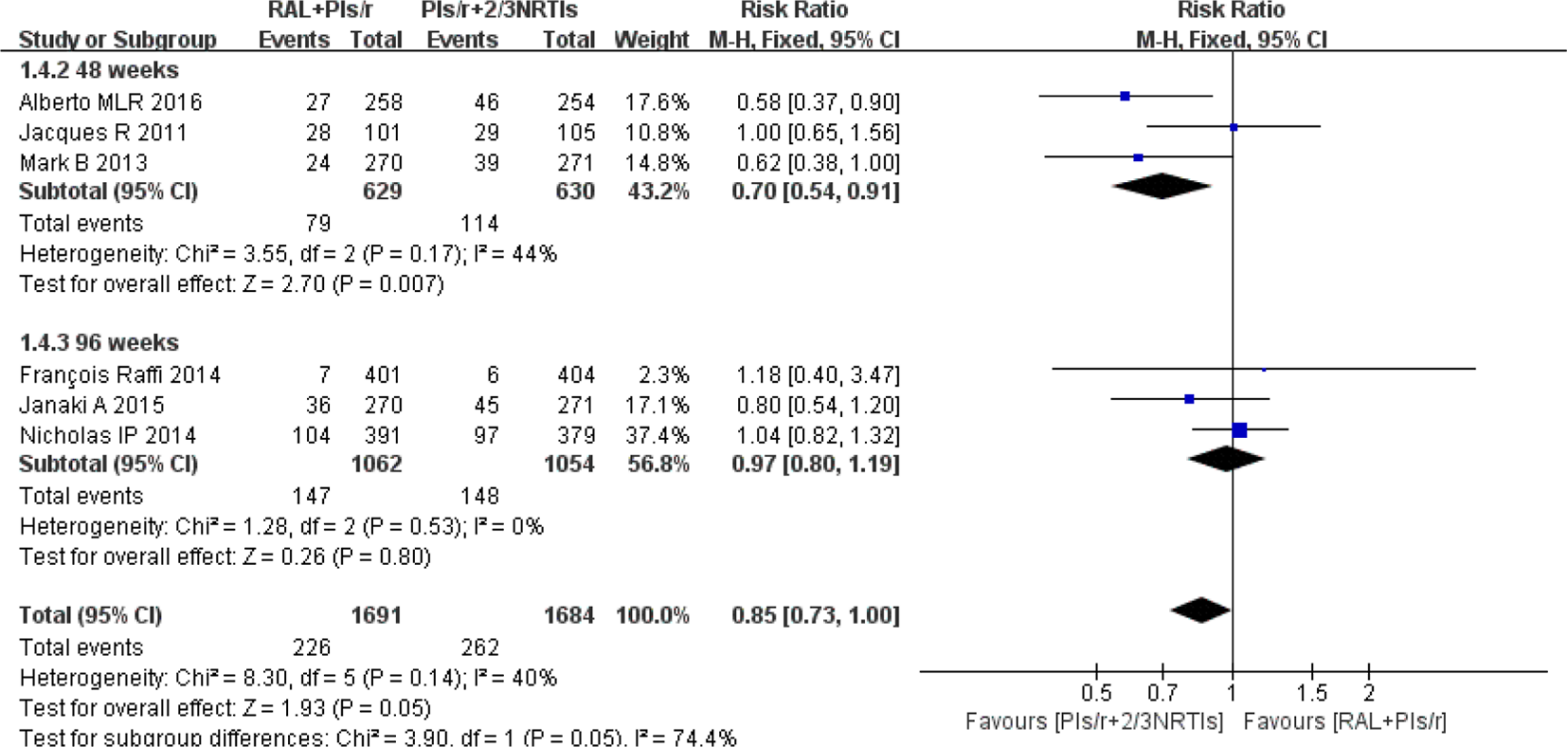
Forest plot comparing grade 3 or 4 adverse events with the RAL-based simplified regimen and the PIs/r-based traditional regimen in HIV-1 patients. (1) RAL = raltegravir. PIs/r = ritonavir-boosted protease inhibitor. NRTI = nucleoside or nucleotide reverse transcriptase inhibitor. (2) Study item displayed as the first author with the publication year. (3) I^2^ and P are the criteria of the heterogeneity test, with the —◆—pooled odds ratio, —¦— odds ratio and 95% confidence interval.

Regarding adverse events, we chose studies that reported grade 3 or 4 adverse events. The investigators found no significant differences in adverse events between the DT and TT groups at 96 weeks [risk ratio 0.97, 95% CI (0.80, 1.19), P = 0.80] (**Figure 5**). At 48 weeks, there were significantly fewer adverse events in the DT group than in the TT group (risk ratio 0.70, 95% CI [0.54, 0.91], P < 0.01).

#### Drug Resistance

Drug resistance mutations were reported in eight studies. Seven (Reynes et al., 2011; Kozal et al., 2012; Group et al., 2013; Paton et al., 2014; Raffi et al., 2014; La Rosa et al., 2016; Hakim et al., 2018) studies reported drug resistance in patients who experienced virologic failure. Among these studies, we found that three studies reported that drug resistance mutations were associated with PIs/r in patients who failed antiretroviral therapy. Drug resistance was mainly found to occur for RAL and NRTIs. In addition, relevant mutations were found at Q148R/Q, T97T/A, 155H, 143R, and 140S+148H for RAL and at M184V, 70R, 67N, and 65R for NRTIs. Detailed information is shown in **Table 2**.

**Table 2:** Effects of the simplified and traditional treatment regimens on drug resistance.

Study name	Mutation site			Description
	RAL	PIs/r	NRTIs	
Kozal et al. (2012)	Q148R/Q, T97T/A, N155H	Not given	Not given	Of the patients who failed antiretroviral therapy: 36.3% (4/11) of patients were found to have an RAL-resistant mutation; 9.1% (1/11) of patients developed phenotypic resistance to RAL without any evidence of an RAL-drug-resistant mutation; No patient had ATV or NRTI resistance (genotypic or phenotypic).
Reynes et al. (2011)	G163R, N155H, T97A	Not given	M184V, N155H	3.96% (4/101) LPV/r + RAL and 2.86% LPV/r + TDF/FTC (3/105) met the criteria for resistance testing of virologic failure.
Paton et al. (2014)	143R, 155H, and 140S+148H	82A, 46I and 76V	70R, 67N, and 65R	In the patients who failed antiretroviral therapy, there was intermediate- or high-level resistance in 2% of patients in the NRTI group and 1% in the RAL group.
Amin et al. (2015)	Not given	Not given	Not given	Not given
Group et al. (2013)	T66A, T143A/T143C/T143H, A 155H	Not given	M184V, 69 insertion complex 151 insertion complex	Of the patients who failed antiretroviral therapy, RAL and NRTI resistance was found in 14.9% and 14.0% of patients, respectively. No PI mutations were recorded.
La Rosa et al. (2016)	T66A, T97A, T143C/A, A155H	M46I, L76V, V82A	M184V, TAMS, T69A	13.3% (6/50) of participants showed NRTI-resistant mutations; 15.56% (7/50) of participants showed PI-resistant mutations; 26.09% (12/46) of participants showed RAL-resistant mutations
Raffi et al. (2014)	A155H	Not given	L65A	Of the patients who failed antiretroviral therapy, 17.2% (5/29) had resistance to integrase, and 3.4% (1/29) had resistance to NRTI.
Hakim et al. (2018)	Y143R, N155H, Q148H, T97A,	46I/L54V,	70R, 67N, 215Y, 41L, TAM1, 151M	3% (10/321) of participants (viral loads <1000 copies/mL) showed NRTI-resistant mutations 2% (7/321) of participants (viral loads <1000 copies/mL) showed PI-resistant mutations; 7% (10/321) of participants (viral loads <1000 copies/mL) showed RAL-resistant mutations

RAL, raltegravir. NRTI, nucleoside or nucleotide reverse transcriptase inhibitor. PIs/r, ritonavir-boosted protease inhibitor.

#### Adherence and Mortality

**Figure 6:**
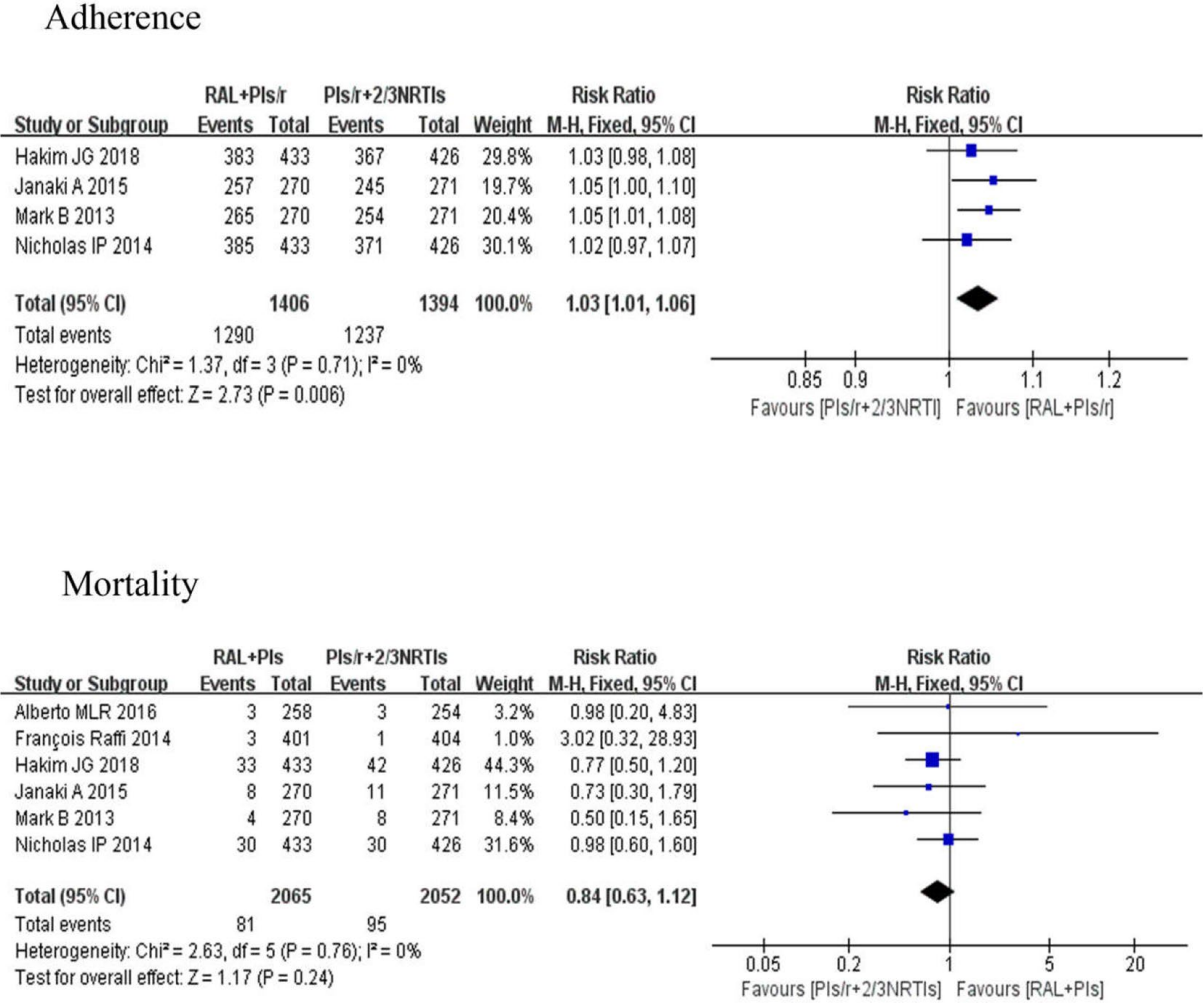
Forest plots comparing adherence and mortality between the two regimens. (1) RAL = raltegravir. PIs/r = ritonavir-boosted protease inhibitor. NRTI = nucleoside or nucleotide reverse transcriptase inhibitor. (2) Study item displayed as the first author with the publication year. (3) I^2^ and P are the criteria of the heterogeneity test, with the —◆—pooled odds ratio, —¦— odds ratio and 95% confidence interval.

Poor adherence is one of the important reasons for antiretroviral therapy failure, and it is also one of the important factors for drug resistance. Four studies (Group et al., 2013; Paton et al., 2014; Amin et al., 2015; Hakim et al., 2018) evaluated adherence. The investigation of adherence was mainly in the form of an adherence questionnaire. There were significant differences in adherence between the DT and TT groups [risk ratio 1.03, 95% CI (1.01, 1.06), P < 0.01]. Six studies (Group et al., 2013; Paton et al., 2014; Raffi et al., 2014; Amin et al., 2015; La Rosa et al., 2016; Hakim et al., 2018), which involved 4,117 participants, reported comparisons of the mortality between two different groups. The risk ratio of mortality in the two groups at the last visit was 0.84 (95% CI 0.63–1.12), and there were no significant differences between any regimens (P = 0.24) **(** **Figure 6** **).**

## Discussion

Until recently, antiretroviral therapy has typically consisted of two reverse-transcriptase inhibitors and a PI/r or an NRTI for treatment-naive and treatment-experience people living with HIV/AIDS (PLWHA) (Boyd, 2011). For maintaining long-term suppressive therapy with the best quality of life, a relatively simple and tolerable regimen is needed (Amin et al., 2015). Drug resistance threatens the long-term efficacy of ART in both developed and developing countries and has led to the development of a new class of drugs termed integrase inhibitors (Wainberg et al., 2011; Whitney et al., 2011; Gupta et al., 2012). RAL, which suppresses the RNA replication of HIV-1 strains, appeared on the market in 2007 and has quickly become a staple of the anti-HIV-1 drug arsenal. The clinical use of RAL represents a milestone that appeared 10 years after ART was introduced to treat AIDS. A PI/r plus RAL was included as an alternative regimen in the 2016 WHO treatment guidelines (WHO, 2016). The WHO-preferred PI/r are atazanavir, lopinavir, and darunavir on the basis of efficacy and tolerability (WHO, 2013a).

This meta-analysis of eight RCTs involving 4,327 PLWHA assessed the clinical value of the effect of DT group versus TT group. All of the studies were based on patients showing a posttreatment plasma HIV-1 RNA viral load <50 copies/mL. According to the viral suppression outcome, DT group is noninferior to TT group at 48 and 96 weeks, whereas the DT regimen was superior to the TT regimen at 24 weeks. The cause of this may be the patient drug sensitivity and high adherence. For the CD4 cell counts, the DT was superior to the TT at 48 weeks and 96 weeks. This indicated that DT group was better than TT group in terms of immunological reconstitution. While there was no clinical significance, the change in CD4 cell counts was 17.6 cell/mm^3^ and 26.5 cell/mm^3^ higher in the DT group than in the TT group at 48 weeks and 96 weeks, respectively. Grade 3 or 4 adverse events resulting from drug regimens have always been a primary focus. In this study, we found no significant difference in adverse events between the DT and TT groups at 96 weeks. At 48 weeks, the DT group had fewer adverse events than the TT group (P < 0.01). The common adverse events in the DT group were fever, skin rash, neurological events, and headache (Reynes et al., 2011; Paton et al., 2014; La Rosa et al., 2016; Hakim et al., 2018). Clinicians should monitor these adverse events and treat them in real time to reduce the interruption of treatment due to adverse events. With respect to drug resistance, the relevant RAL mutation sites were mainly N155H, Q148H/K/R, G140S, Y143R, and T97A (Reynes et al., 2011; Kozal et al., 2012 Group et al., 2013; Paton et al., 2014; Raffi et al., 2014; La Rosa et al., 2016; Hakim et al., 2018). A patient may develop resistance with the occurrence of any two mutations. Three studies (Paton et al., 2014; La Rosa et al., 2016; Hakim et al., 2018) mentioned PI (LPV)-resistance mutation sites, which were M46I, L76V, and V82A. In our review, we found that, despite the presence of mutations, there were few patients who failed ART due to RAL or PIs resistance. However, multidrug combinations and the pill burden of such programs will lead to poor treatment compliance issues. Our review found that RAL plus PIs/r could enhance the adherence of patients. To a great extent, reducing the number of patients with poor adherence and antiretroviral failure may prolong the life of patients. At the same time, drugs with a high genetic barrier can be used as the main treatment to avoid premature cessation due to drug resistance and untimely treatment. Although RAL exhibits rapid, efficient, and long-lasting antiretroviral activity, it is expensive and is currently not included on the list of free antiretroviral drugs in China; as such, RAL is bound to be a heavy economic burden to most AIDS patients.

Our study has some limitations. First, the number of included studies in our review was too low. The analysis of some important outcomes, including mortality and the number of and reason for discontinuations, was limited by a low number of events. Second, the varying numbers of participants brought some uncertainty to the research results. The statistical analysis will incorporate a certain deviation, resulting in result errors. Finally, the studies were from various settings, including low-income, middle-income, and high-income countries; most were from low-income countries. Although this is an external factor, we cannot ignore the internal effects of external factors.

Increasing attention has been directed toward the development of drugs for PLWHA who have failed antiretroviral therapy. This meta-analysis indicates that combinations of RAL with PIs/r not only decrease the plasma HIV-1 RNA load and enhance the CD4 cell counts but also result in fewer side effects. Additionally, there was no obvious drug resistance to these combinations. In summary, RAL combined with PIs/r could be a beneficial and safe therapeutic choice for PLWHA, and higher adherence rates and realization of immune reconstruction could improve quality of life and enhance patient preference; this strategy is worthy of clinical promotion.

## Author Contributions

HW, XH, and YH conceptualized the study and developed the research protocol. YH identified articles for full-text review and extracted the data that matched the inclusion criteria. YH and HC performed the statistical analyses. All authors contributed to the writing of the manuscript. YC and XH polished and revised the manuscript.

## Conflict of Interest

The authors declare that the research was conducted in the absence of any commercial or financial relationships that could be construed as a potential conflict of interest.
